# Neurotransmitter and Intestinal Interactions: Focus on the Microbiota-Gut-Brain Axis in Irritable Bowel Syndrome

**DOI:** 10.3389/fendo.2022.817100

**Published:** 2022-02-16

**Authors:** Minjia Chen, Guangcong Ruan, Lu Chen, Senhong Ying, Guanhu Li, Fenghua Xu, Zhifeng Xiao, Yuting Tian, Linling Lv, Yi Ping, Yi Cheng, Yanling Wei

**Affiliations:** ^1^ Department of Gastroenterology, Daping Hospital, Army Medical University (Third Military Medical University), Chongqing, China; ^2^ Department of Pathogenic Biology and Immunology, School of Basic Medicine, Ningxia Medical University, Yinchuan, China

**Keywords:** neurotransmitters, irritable bowel syndrome, microbiota-gut-brain axis, 5-HT, dopamine, GABA, histamine

## Abstract

Irritable bowel syndrome (IBS) is a functional gastrointestinal disorder of unknown etiology. IBS is caused by a disruption in the gut-brain axis. Given the importance of the gut microbiota in maintaining local and systemic homeostasis of immunity, endocrine, and other physiological processes, the microbiota-gut-brain axis has been proposed as a key regulator in IBS. Neurotransmitters have been shown to affect blood flow regulation, intestinal motility, nutrient absorption, the gastrointestinal immune system, and the microbiota in recent studies. It has the potential role to play a function in the pathophysiology of the gastrointestinal and neurological systems. Transmitters and their receptors, including 5-hydroxytryptamine, dopamine, γ-aminobutyric acid, and histamine, play an important role in IBS, especially in visceral sensitivity and gastrointestinal motility. Studies in this field have shed light on revealing the mechanism by which neurotransmitters act in the pathogenesis of IBS and discovering new therapeutic strategies based on traditional pharmacological approaches that target the nervous system or novel therapies that target the microbiota.

## Introduction

Irritable bowel syndrome (IBS) is a chronic functional gastrointestinal disorder (FGID) and one of the most prevalent gastrointestinal diseases. The pathogenesis of IBS is multifactorial, includes genetic, physiological, psychosocial, and environmental factors. The clinical characteristics of IBS present as persistent or intermittent episodes, including abdominal pain, abdominal distention, bowel habits, and changes in stool behavior, thus severely affecting the quality of life (1, 2). Meanwhile, patients with IBS often have neurological dysfunctions, such as anxiety, depression, and other symptoms (3). However, due to its complexity, the underlying mechanisms of IBS pathogenesis are still a mystery (4). Currently, it is believed that the etiology of IBS may involve mental disorders, gut dysbiosis, gastrointestinal motility disorders, visceral hypersensitivity, and intestinal infection (5).

**Figure 1:**
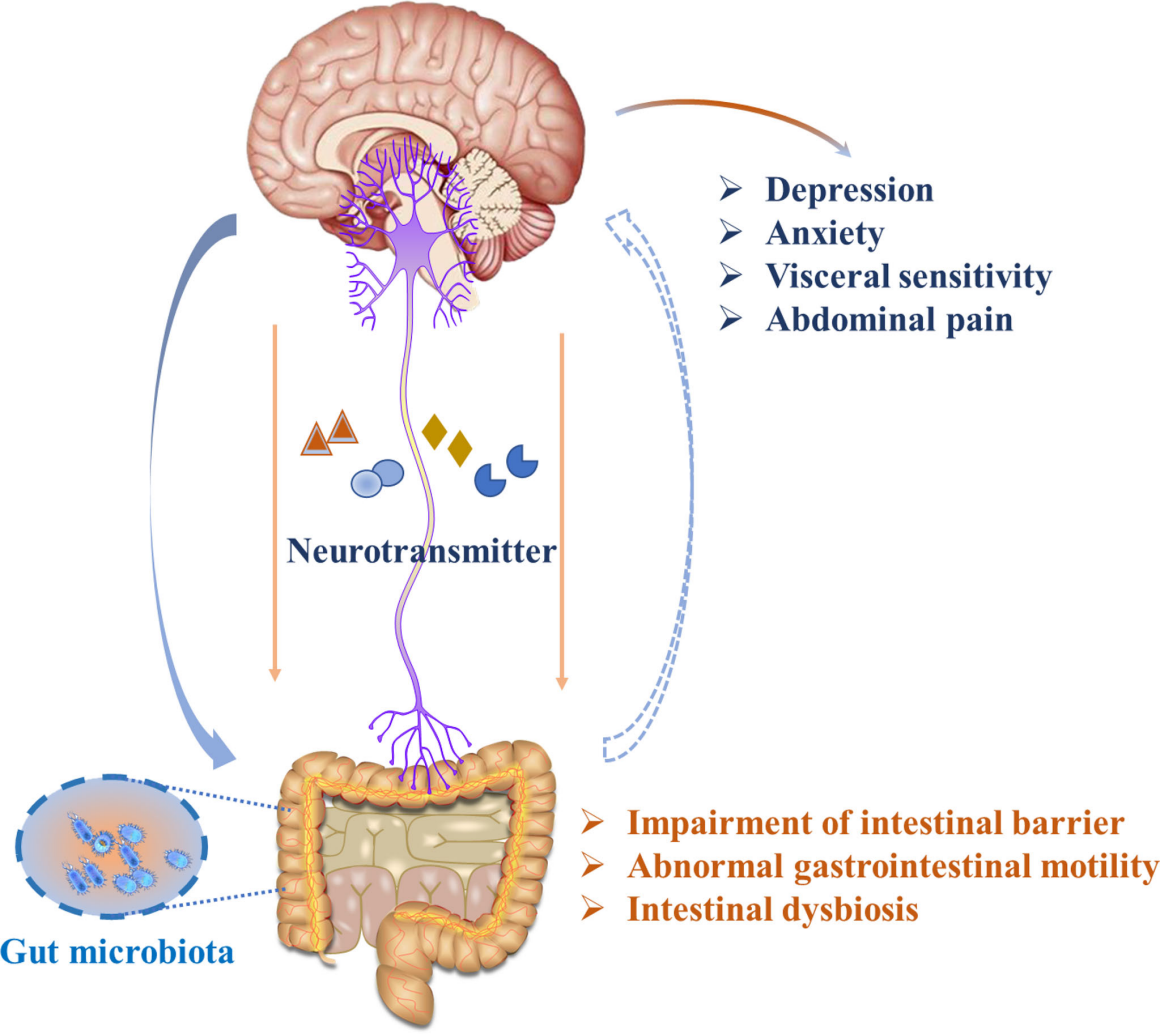
Illustration of gut-brain bidirectional communication in IBS.

Several mechanisms have been proposed in IBS pathogenesis, including abnormal neural pathways and alterations in the immune and endocrine systems, Together, these elements lead to malfunctions in regulating intestinal smooth muscle movement (**Figure 1**). Notably, recent studies highlight that the gut microbiota plays a role in inflammation and immune dysfunction *via* the gut-brain axis, which may contribute to IBS pathophysiology (6). At the same time, a series of clinical and animal studies showed that the abundance of some dominant microorganisms was decreased, gut microbiota diversity was decreased in IBS individuals, and the abundance of *Bifidobacteria* and *Lactobacilli*, as well as *Enterobacteria* was increased. Moreover, it has also been reported that host resistance to pathogenic microorganism colonization is weakened in patients with IBS. Many elegant works have proposed that IBS is likely to be caused by intestinal flora imbalance, which is also related to the induction of an abnormal neuroendocrine network. The imbalance of intestinal flora can lead to impaired intestinal mast cell function. Intestinal mast cells play an important role due to their irreplaceable functions in the intestinal mucosal immune system and nervous system. The inflammatory mediators secreted by intestinal mast cells act on adjacent endocrine cells and nerve fibers to release neurotransmitters, which affect intestinal motility, and sensation and can transmit information to the nerve center, inducing high sensitivity of the visceral afferent nerve, the intestinal nerve, and the intestinal nervous system, resulting in intestinal dysfunction and IBS symptoms (7). However, the mechanism by which the intestinal flora plays a role is not fully understood. With the increase in histamine and protease levels in the colon biopsy supernatant of patients with IBS, human submucosal neurons were excited (8). Changes in stress hormones and brain-derived neurotrophic factor (BDNF) levels occurred, but it seems that more neurotransmitter systems or regulators may be the basis for microbial changes in host behavior, and there was a lack of insight into these pathways at the time (9, 10). Moreover, our previous studies also focused on the topic of the “microbiota-gut-brain axis”, and an open-label clinical study proved that children with autism spectrum disorders had a significant improvement in gastrointestinal as well as autistic symptoms after being given fresh feces by colonoscopy and fecal microbiota transplantation (FMT) capsules (11).Additionally, an animal study showed that ulcerative colitis (UC) animal models exhibited depressive symptoms, and rectal administration of the probiotic *Roseburia intestinal* is helpful in colon repair and the recovery of gastrointestinal function by restoring the gut microbiota (12), These alterations in gastrointestinal malfunctions are also followed by the alleviation of depressive-like behaviors through the gut-brain axis.

**Figure 2:**
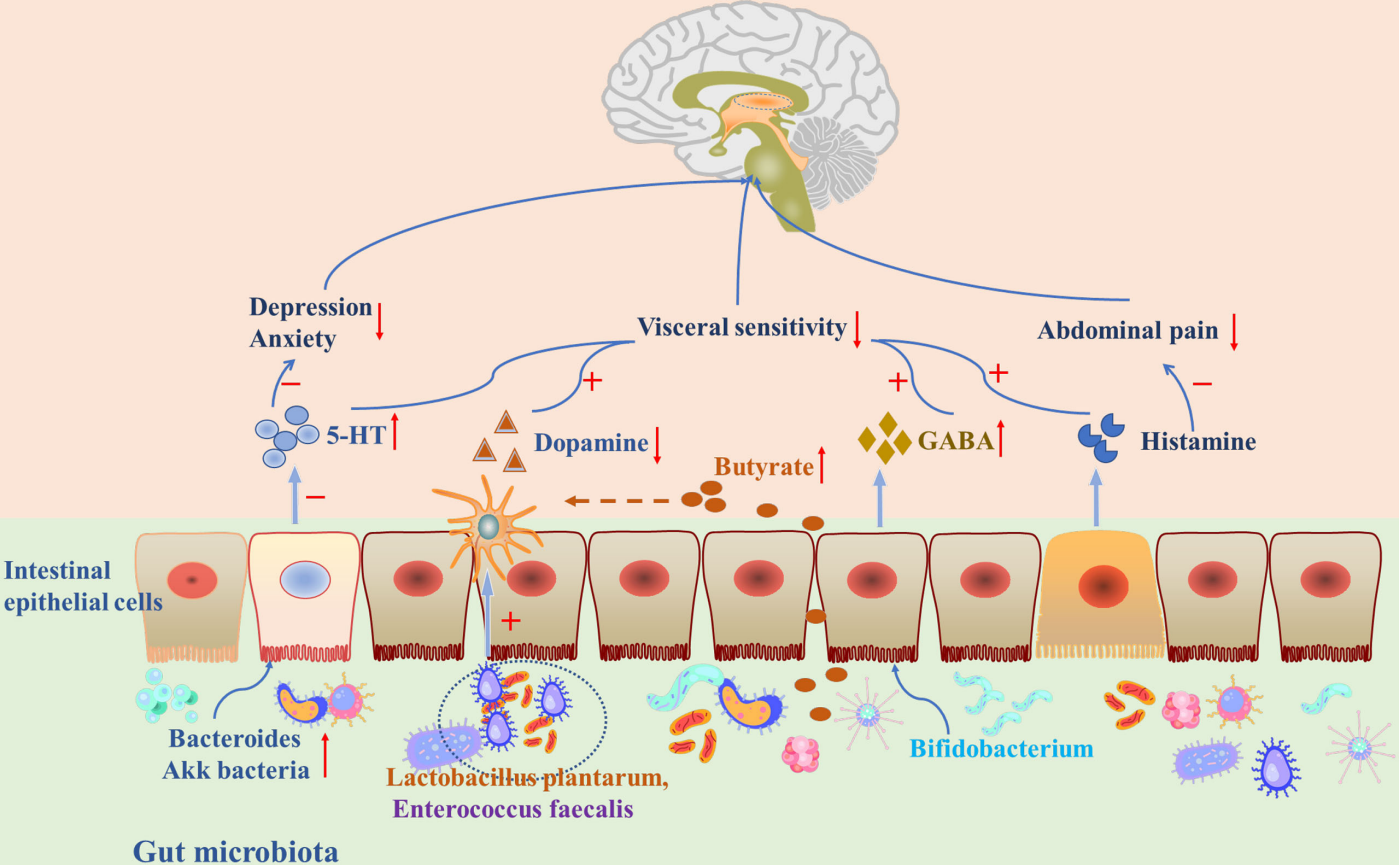
Gut microbiota and neurotransmitters played an important role through the microbiota-gut-brain axis in IBS.

Currently, available evidence has shown that neurotransmitters in the gastrointestinal system might be important in regulating the microbiota-gut-brain axis in IBS. This review will focus on the possible role of neurotransmitters including 5-hydroxytryptamine (5-HT), dopamine, γ-aminobutyric acid (GABA), and histamine in the microbiota-gut-brain axis (**Table 1**), and aims to summarize recent clinical and animal studies to explain how neurotransmitters are involved in FGIDs through synthetic mechanisms, gastrointestinal distribution, and therapeutic targets (**Figure 2**).

**Table 1:** Research on neurological disorders and the microbiota-gut-brain axis.

Neuroticism	Location	Gastrointestinal Function (Constipation or diarrhea. etc.)	Neurological diseases with gastrointestinal dysfunction	Gut bacteria disorder	Clinical medication
**5-HT**	Enterochromaffin cells (ECs), mucosal mast cells, and myenteric neurons (13–15)	Diarrhea (16, 17) Abdominal pain and discomfort (16)	Affective disorders (18) Multiple sclerosis (19) Major Depressive Disorder (20)	Indigenous spore-forming bacteria (Sp) (21)	Ondansetron (22) Tricyclic antidepressants (TCA) and selective serotonin reuptake inhibitors (SSRIs) (18)Resveratrol (23)
**Dopamine**	Nerve terminal layer of the intestinal wall, and the intestinal mucosa (24)	Visceral pain Increase intestinal permeability (25, 26)	Anxiety (27, 28) Depression (29, 30) Multiple sclerosis (31) Schizophrenia (32), Alzheimer’s disease (AD) (33) and Parkinson’s disease (PD) (34)	*Enterococcus faecalis* (35) *Lactobacillus plantarum* PS128 (36)	Metformin (25) Butyrate, Losartan (26) Imipramine (37)
**GABA**	In intermuscular and submucosal neurons and intestinal epithelial cells (38)	Intestinal motility, gastric emptying, nociceptive sensation, and acid secretion (39)	Behavioral disorders, pain, and sleep (40, 41) Major Depressive Disorder (42)	*B. fragilis* KLE1758 (42)	Pregabalin, gabapentin or baclofen (43)CGP7930 (39) *Bifidobacterium* NCIMB8807 (44)
**Histamine**	Gastrointestinal chromaffin cells (45)	Gastric acid, gastrointestinal inflammation, and abdominal pain (46)	Major Depressive Disorder (47)	*Plesiomonas shigelloides Streptococcus thermophilus*, *Staphylococcus warneri*, *Lactobacillus parabuchneri*, and *Lactobacillus reuteri* (48)	Ebastin (49) Ketotifen (50)

## Irritable Bowel Syndrome

The pathological mechanism of IBS is unclear, and some studies have reported that it may be related to psychology, central nervous modulation, neuroendocrine response, gastrointestinal motility, visceral hypersensitivity, etc (51). The recently revised Roman standard IV defines irritable bowel syndrome as “In the past three months, abdominal pain occurred on average at least one day per week, accompanied by two or more of the following symptoms: i) related to a change in defecation frequency, ii) associated with defecation, iii) related to a change in stool form (consistency); symptoms should last for at least six months” (52). Roman IV-based questionnaires on bowel habits and abdominal pain are widely used to diagnose IBS and further determine IBS subtypes based on bowel movements and stool conditions. According to clinical manifestations, IBS can be classified into the following subtypes: unclassified (IBS-U), mixed type (IBS-M), constipation-predominant (IBS-C), and diarrhea-predominant (IBS-D) (52, 53), among which diarrhea type is the most prevalent. The gut microbiota of patients differs among different IBS subtypes, and it is speculated to be related to the typical constipation subtype (54). Studies have reported that patients with reduced IBS symptoms after FMT had lower depression scores, but the depression scores of patients who received placebo treatment did not change (55).

From the above evidence, IBS is generally thought to be a multifaceted disease with a combination of peripheral and central factors. In recent years, the conception of the microbiota-gut-brain axis has been proposed, and the gut microbiota has been identified as an indispensable participant in gut-brain communication (56). For the intestinal microbiota hypothesis, the microbiota can influence the brain and behaviors along the microbiota-gut-brain axis (57). Recent studies have reported that IBS-related mental disorders (including schizophrenia, anxiety disorders, and depression) are related to or regulated by changes in the microbiota, while probiotics and antibiotics, exogenous probiotics, and microbial substrates have a certain therapeutic effect on these symptoms (58). A series of data from animal experiments indicate that the gut microbiota may affect the brain and even lead to neurological and mental illness. Therefore, microbiota may become a potential indicator or a therapeutic target of many mental diseases, such as depression, Parkinsonism, and IBS (59).

## Neurotransmitters

Neurotransmitters (NTs) are chemical messengers that transfer a message from one neuron to the next, they are essential for neurological functions and influence human behavior. Mental disorders such as depression, anxiety, and mood disorders are closely related to the abnormalities in neurotransmitters (60). The monoaminergic neurotransmitter deficiency hypothesis suggests that joy, happiness, and other positive emotions are associated with 5-HT, norepinephrine (NE), and dopamine, while inadequate neurotransmitter levels would result in depressive symptoms. Restoring the levels of these neurotransmitters will have antidepressant effects (61). Subsequent studies have shown that signals from other neurotransmitters may also change during depression. For example, glutamate and acetylcholine neurotransmitter levels are higher, while GABA neurotransmitter levels are lower (60, 62, 63).

Neurotransmitters are produced not only by host cells, but also by the intestinal flora, hence, gut microbes also affect the central nervous system (CNS) through the microbiota-gut-brain axis (64, 65). A recent study revealed that changes in the gut microbiota may contribute to mutual communication between the brain and the intestine, and may change the cortical response through neuroendocrine-immune stimulation (66). For example, it is believed that the pathogenesis of depression may be related to the gut microbes, which are indispensable in short-chain fatty acid (SCFA) metabolism and play a crucial role in regulating neurotransmitters in the CNS, especially in the hypothalamus (67).

In IBS patients, the physiological manifestations are closely related to neurotransmitters, including abnormal gastrointestinal motility, visceral sensory abnormalities, central sensory abnormalities, anxiety, and depression (68). Changes in microbial composition and metabolomics are related to the abnormality in neurotransmitter expression in the system, and would also influence the intestinal neuronal pathways that control intestinal sensorimotor function (69, 70) These neurotransmitters not only regulate blood flow, but also influence intestinal movement, the absorption of nutrients, natural immunity of the gastrointestinal system, and the microbiota. Therefore, the pathways and mechanisms that are involved in neurotransmitter synthesis, as well as neuron inactivation, could serve as potential targets for therapeutic drugs for psychiatric and neurological diseases because they play such an important role in brain function.development (71). In this review, we will mainly discuss the interaction of neurotransmitters and the intestine through the microbiota-gut-brain axis in IBS.

### 5-HT

5-hydroxytryptophan (5-HT) is a type of indoleamine [3-(b-aminoethyl) -5-hydroxyindole]. It serves as not only a systematic neurotransmitter and a hormone in the body, but also a paracrine messenger in the gastrointestinal system (72). 5-HT in the body is synthesized from the essential amino acid tryptophan (Trp) in both the brain and gut, and the majority of it (approximately 95% of total 5-HT) resides in the digestive tract and is mostly produced and stored in enterochromaffin cells (ECs) (13, 73). ECs are the most characteristic endocrine cells in the intestine,—the largest endocrine organ in the human body, —and can transform tryptophan to 5-HT *via* Trp hydroxylase 1 (TPH1). Peripheral 5-HT plays an essential role in regulating intestinal sensation, movement, secretion of intestinal glands, and upholding intestinal balance, it does not cross the blood-brain barrier and affects the CNS in healthy conditions. In the human intestine, ECs act as sensors for the gut content. Under certain stimulations, including acetylcholine, increased intracavitary pressure, and low pH (74–76), 5-HT is released from ECs and activates the intrinsic sensory neurons in the intestinal wall to cause secretory reflex and peristalsis in the intestine, while exogenous neurons can also be activated by 5-HT to cause pain, discomfort, nausea, and vomiting (75, 77, 78)

In the gut, 5-HT is responsible for one of the core signaling pathways, especially in modulating intestinal permeability and regulating mucosal inflammation. In particular, intestinal peristalsis and the secretory reflex are regulated by 5-HT released from endothelial cells. They are stimulated by 5-HT_1_ and 5-HT_4_ receptors on submucosal primary afferent neurons, while 5-HT_3_ receptors on primary afferent neurons may also play a part in intestinal reflex activity (13). When the intestinal tract is stimulated, 5-HT increases and binds to 5-HT_3_ receptors in the exogenous primary afferent nerve endings, making the enteric nervous system (ENS) and visceral afferent nerve highly sensitive, thus resulting in discomfort, abdominal pain, and diarrhea (16). 5-HT is related to colon inflammation in a DSS-induced murine colitis model, and treatment by inhibiting 5-HT production in colonic mucosa has a therapeutic outcome in ameliorating colitis-associated symptoms and inflammation (79). In addition, it has also been reported that in IBS-D patients, the 5-HT and 5-HT_3_ receptors in the intestinal mucosa are significantly higher than those in healthy controls, indicating an impaired 5-HT system in IBS patients (80).

5-HT production in the gut is believed to be regulated by the gut microbiota, although the mechanism by which the gut microbiota acts in modulating intestinal 5-HT synthesis has not yet been fully illustrated. Efforts have been made to understand how the gut microbial community affects Trp metabolism and 5-TH. Alterations in gut microbial composition can lead to changes in the plasma level of Trp along with its metabolites (79). Transplantation of feces from IBS-C patients to healthy mice would cause gut microbiota imbalance by reducing *Firmicutes* and increasing *Bacteroides* and *Akkermansia*. Additionally, the mice showed a reduction in 5-HT in the intestinal tissue, and therefore suffered from constipation and damaged intestinal barrier function (81). Germ-free mice exhibit colon defects in producing 5-HT as well as decreased serum 5-HT levels, and current evidence suggests that SCFAs produced by the gut microbiota may play a role in regulating TPH1 expression (82). Moreover, secondary bile acids oriented from cholate *via* microbial transformation can influence the synthesis of 5-HT (21). In addition, 5-HT is capable of increasing the abundance of spore-forming bacteria in the gut microbiota (83).

By focusing on 5-HT and the gut microbiota, traditional Chinese medicine has made a breakthrough in treating IBS. Tongxie prescription can effectively rebuild the gut microbiota and gut microecology, reduce colonic 5-HT, and thus relieve the symptoms of IBS-D (84). The oral administration of resveratrol, an effective antioxidant, had a strong therapeutic effect on IBS rats through the 5-HT1A receptor-dependent PKA-CREB-BDNF pathway, and changed the concentration and metabolic rate of 5-HT, as well as the expression of its receptor 5-H1A (23). In a work that reports the beneficial effects of ondansetron, a 5-HT_3_ receptor antagonists, on IBS-D patients who showed significant abnormalities in mucosal serotonin metabolism, the therapeutic effect of ondansetron was most obvious in patients with the lowest 5-HT concentration in rectal biopsy tissues (22), and the possible mechanism may be that 5-HT_3_ receptor antagonists slow down the intestinal transport. In addition, a meta-analysis reported that 5-HT_3_ receptor antagonists are effective in improving symptoms in IBS-D (85, 86). Other studies have proven that blocking the 5-HT_6_ receptor can reduce the number of defecations, and the use of 5-HT6 receptor antagonists can relieve symptoms of IBS-D or other forms of diarrhea (17).

### Dopamine

Dopamine is a major catecholamine neurotransmitter that regulates reward-motivated behavior, and it is synthesized by both the central and peripheral nervous systems. It is also a precursor of other catecholamines such as NE and epinephrine. Dopamine is pivotal in multiple physiological processes, including attention, motivation, reward, emotion, memory, and appetite (87, 88). Most dopamine originates from tyrosine, phenylalanine hydroxylase can convert L-phenylalanine into tyrosine and it can also indirectly produce dopamine (87, 89). Although dopamine along with its terminal products can be detected in the cerebrospinal fluid and blood, it is difficult to trace its source origin due to its complexity, it can be produced not only by the CNS but also by peripheral organs such as the kidneys and intestines (87).

Interestingly,mutual modulation exists between microorganisms and catecholamines. In the presence of dopamine and NE, pathogenic *Escherichia coli* O157:H7 (EHEC) displayed higher viability, increased motility, and stronger virulence. Other pathogenic bacteria such as *Klebsiella pneumoniae* and *Staphylococcus aureus* were also found to be sensitive to NE, which is possibly caused alterations in iron acquisition. In addition, some strains of gut microorganisms are reported to be capable of producing dopamine or NE, including *Proteus vulgaris*, *Serratia marcescens*, *Bacillus subtilis*, and *Bacillus mycoides (* 90). Meanwhile, dopaminergic circuits have also been proven to be sensitive to gut microbiota alterations. Some studies report that germ-free (GF) and specific pathogen-free (SPF) mice showed anxiety behavior under different stresses, with no significant change in dopamine levels in the hippocampus (45, 91). Diaz Heijtz et al. reported that the ratio of dihyroxy-phenyl acetic acid and dopamine (DOPAC/DA ratio) in the striatum was significantly higher in GF mice. Nishino and his group, found that fecal microbiota transplantation from SPF mice to GF mice could relieve anxiety symptoms in GF mice. Strikingly, the most significant change found in monoamine in GF mice after fecal transplant is the alteration in dopamine and its metabolites.

A study on GF mice also reported that some probiotics have positive influences on anxiety behavior, *Lactobacillus Plantarum PS128* showed an anti-anxiety effect, while dopamine and homovanillic acid (one of the catecholamine metabolites) were higher in the striatum, but there were no significant changes in the prefrontal cortex, hippocampus, and striatum (36). Recently, dopamine has been considered to be related to intestinal homeostasis. Dopamine receptors which are distributed in the intestinal wall (D1, D3, and D5 receptors) are present not only in the intestinal mucosa but also in the nerve endings of the intestinal wall. Dopamine D2 receptors are the main mediators of the endogenous effect of dopamine (71, 92), they are found only in nerve endings in the intestinal wall, while the D_4_ receptor exists only in the mucosal layer (24).

Dysregulation of the dopaminergic system is associated with anxiety (27), depression (29, 30)and intestinal microbe imbalance (93). Alterations in the dopaminergic system are found in patients with IBS, compared with the healthy cohort, the IBS cohort has lower serum and urinal levels of dopamine (22, 94). However, another clinical study reported that dopamine levels were significantly increased in IBS-C patients, while IBS-D patients did not show any difference (95). These opposite results are unable to reflect the real-world situation of how dopamine and dopamine-related metabolism is changed in IBS patients, but evidence validated that dopamine could contribute to IBS. Some researchers have made efforts to develop dopamine-based therapy for IBS, and they have gained progress on this issue. In restless legs syndrome (RLS) cases associated with IBS, the administration of levodopa and dopamine agonists induced improvement in both IBS and RLS symptoms (96). It has also been reported that metformin, a widely used drug for treating type 2 diabetes, can activate central D_2_ dopamine receptors, thus reducing visceral hypersensitivity and increasing intestinal permeability in IBS patients (25),. Similarly, butyrate enema can improve visceral pain and colon permeability in an IBS animal model. Losartan can prevent visceral pain and colonic hyperpermeability in IBS rats, which may be dependent on the PPAR- γ, AMPK, and central dopamine D_2_ pathways, or mediated by opioids and nitric oxide (26). Imipramine may also inhibit visceral hypersensitivity and colonic permeability in an IBS animal model through the α-2-adrenoceptor, dopamine D_2,_ and opioid pathways, and improve the intestinal barrier (37).

### Gamma-Aminobutyric Acid

Gamma-aminobutyric acid (GABA) is an amino acid derivative of glutamate, which is a major mediator of inhibitory transmission in the mammalian nervous system. A large amount of literature supports the association between changes in GABA neurotransmission and many psychological diseases, including behavioral disorders, insomnia, and pain (40). GABA also plays an important role in homeostasis and disruption of the ENS, such as acid secretion, gastric empties, intestinal motility, and pain perception (39, 90). The sources of GABA in the intestine include neurons containing GABA synthase, and mucosal endocrine-like cells, indicating that GABA is not only a neurotransmitter but also an endocrine agent in the gastrointestinal tract (97). In IBS patients, the GABAergic system is disrupted, and the levels of glutamate decarboxylase 2 (GAD2), GABA, and GABA receptors(including type B1 and B2) are decreased, while GABA transporter-2 (GAT-2) is increased in IBS-D patients (98).

For years, evidence has shown that bacteria are responsible for GABA production as well as consumption. The *E. coli* strain was found to be capable of taking GABA as the only source of carbon and nitrogen decades ago, while a broad spectrum of bacteria was been identified as GABA producers (90), including *Bifidobacterium* and *Lactobacillus*. The gut microbiota has a direct influence on GABA metabolism in the body. Fecal transplantation from schizophrenia patients to GF mice resulted in elevated levels of glutamine and GABA in the hippocampus, and led to schizophrenia-related behaviors, which are similar to other glutamatergic hypofunction murine models (99). Meanwhile, compared to SPF mice, GABA levels in the lumen and serum of GF mice are remarkably reduced, while the cerebral level is intact (100). It is well known that dietary intervention can change the composition and function of the gut microbiota (101), and a ketogenic diet has been shown to increase the level of GABA in the cerebrospinal fluid of children with refractory epilepsy (102). In a recent fecal transplant study, it was found that GABA was the most variable metabolite in obese patients receiving allosteric fecal transplants from lean donors (103). Unfortunately, how GABA is produced by gut microorganisms and how it is involved in the disease are still underunderstood.

GABA receptors in the mammalian CNS enable GABA to act as a depressant and influence behavior. GABA-A receptors are the major receptor of inhibitory neurotransmission in the CNS, and are involved in most brain physiological functions (104). Enteric nerve cells exert excitatory effects by increasing intracellular chloride concentrations through sodium-potassium-chloride transporters, thus activating GABA receptors in the ENS, rather than inhibiting neurons in the CNS. The understanding of the GABAergic system in the peripheral nervous system is relatively limited, especially in the ENS. Studies have proven that both ionic (GABA-A and GABA-C) and metabolic (GABA-B) receptors of GABA exist in both nerve cells and nonnerve cells in the GI system (105, 106). The mRNA level of the GABA-A receptor was expressed in intermuscular and submucosal neurons and intestinal epithelial cells (105). Seifi et al. demonstrated immunolocalization of GABA subunits (α1-5 and γ2) on mouse colon ENS cells (107). Other studies have also shown that a nonspecific GABA-A receptor ligand affects intestinal contraction (108, 109).

The fact that the GABA-B receptor changed in IBS patients and the evidence that mice exposed to stress had higher levels of GABA-A receptor α3 in their colons indicated that stress may be the reason for the change in GABA in IBS (110). Therefore, GABA analogs and agonists may be effective in the treatment of IBS. It has been reported that pregabalin may improve IBS symptoms because it binds to calcium channels in ileum neurons (43). In recent years, the US FDA has approved pregabalin for the treatment of fibromyalgia and neuropathic pain because of its analgesic and anxiety-relieving effects. And Clinical studies have proven that pregabalin has a positive effect on IBS symptoms, especially IBS-M and IBS-D. Although pregabalin can improve abdominal distention, abdominal pain, and diarrhea, it does not affect IBS symptoms such as depression and anxiety (111). In addition, gabapentin and baclofen are effective and helpful for relieving visceral hypersensitivity. The pharmacological effects of gabapentin on IBS were limited due to hepatotoxicity and neurotoxicity, but it still improved pain and anxiety-like behavior in mice (112), moreover, baclofen did not show significant efficacy in reducing the visceral motor response (113). CGP7930 is another GABA-BR agonist, and due to its mechanism of action, it reduces visceral pain without the same number of side effects as baclofen and promotes endogenous GABA release (39). Although the GABA receptor is considered to be a potential target in therapies for IBS, it also shows significant side effects. Notably, the influence of GABA on host cells could be enhanced by the microbiota. Oral supplementation with *Bifidobacterium brevis NCIMB8807pESHgadB*, a strain that produces GABA through overexpression of glutamate decarboxylase B, reduced visceral sensitivity in a rat model (44).

### Histamine

Histamine is produced from the amino acid L-histidine by catalyzed oxidation decarboxylation of histidine decarboxylase, which exists in many mammalian cells (87). Most of them are expressed in mast cells and basophils, but can also be detected in lymph nodes, thymus, and gastrointestinal chromaffin cells (45). Histamine is an important regulator in various immune responses, such as allergies and inflammation, and it can also modulate the motility of the gastrointestinal tract, increase the permeability of the intestinal mucosa, and affect mucosal ion secretion. Based on this, histamine is speculated to be involved in IBS pathogenesis. Several clinics and animal studies have found that the histamine level in the colon was increased in IBS cases (114).

The metabolism of histamine mainly depends on histamine-N-methyltransferase (HNMT) and diamine oxidase (DAO) (115, 116). According to its location, histamine *in vivo* is deaminated or methylated by DAO and HNMT enzymes, respectively (117). DAO is an extracellular secretion enzyme that oxidizes and deaminates histamine to produce imidazole acetaldehyde. Animal studies have shown that DAO forms the main hurdles for histamine absorption (118). HNMT is a cytoplasmic enzyme that forms N4-methylhistamine by methylation of inactivated histamine imidazole rings, requiring receptor-mediated endocytosis or specific transporters to transport histamine into cells (119). Furthermore, histamine is synthesized by neurons in the posterior hypothalamus, which extends from axons to the entire brain and acts as a neurotransmitter (120). Apart from host cells, histamine can also be produced by some strains of microorganisms, such as *E. coli* and *Morganell morganii*. Interestingly, certain bacteria are capable of regulating the synthesis of histamine by producing histidine decarboxylase (HDC), an enzyme that transforms histidine to histamine (114).

It was recently proposed that histamine receptor agonists for treating allergies, can also reduce visceral hypersensitivity, immune activation, and symptoms in IBS patients (121). Urinary histamine levels in IBS patients are related to the disease severity of IBS, especially abdominalgia (94). Some studies have shown that histamine-tolerant patients can reduce the α diversity of gut microbiota, change the abundance of *Proteus* and *Bifidobacteria*, and elevate the fecal zonulin level (122). Preclinical and clinical results suggest that pain in some patients with IBS may be caused by fungus-induced mast cell-derived histamine release, which in turn activates sensitization of sensory-afferent expressed histamine-1 receptors and associated nociceptive transient reporting potential channel V1(TRPV1) (123).

Histamine can activate its 7-transmembrane G-protein coupled receptors, including H1, H2, H3, and H4 (45), which are expressed on both presynaptic and postsynaptic nerve membranes (124). Presynaptic histamine receptors, as autologous or heterologous receptors, regulate neurotransmitter release from the axon terminal to synaptic cleft *via* different responses (125). Histamine receptors are distributed in various places of the nervous system, and their specificity of localization depends on their physiological correlation (126). Studies have shown that H1 and H4 are the main histamine receptors involved in the gastrointestinal process, and H2 is associated with the production of gastric acid (46).

IBS patients who respond well to H1 antagonists differ from others, hinting that the histaminergic system of IBS patients may be overstimulated (127). Recent evidence has shown that histamine increases the sensitivity of mouse dorsal root ganglion and human rectal submucosal neurons to TRPV_1_ by activating the H_1_ receptor (HRH_1_). In addition, the supernatant of IBS biopsy tissue also enhanced the sensitization of mouse dorsal root ganglion neurons through HRH_1_. Based on these findings, 51 IBS patients were given ebastin, a nonsedative HRH1 antagonist,and after treatment, ebastin reduced IBS symptoms and abdominalgia in patients (49). Since the expression was increased in the colonic mucosa of colitis-infected, H1 and H4 receptors may have an important effect on the pathogenesis of colitis and visceral hypersensitivity. In a model of postinflammatory colitis, an H_4_ antagonist can improved abdominal pain (128). New interventions to block H_1_ receptors are being proposed because ebastin improved IBS clinical signs such as abdominalgia and visceral allergy (49), while ketotifen can upregulate the pain threshold and improve the quality of life of IBS patients (50).

### Other Neurotransmitters

The abovementioned neurotransmitters involved in IBS *via* the microbiota-gut-brain axis were intriguing, and other neurotransmitters have also attracted attention for understanding the etiology and pathogenesis of IBS. Many researchers have proven that NE and glutamate are responsible for the occurrence of IBS. For example, one study pointed out that NE levels in IBS patients changed after taking the α-2 receptor antagonist yohimbine and agonist clonidine. IBS patients have increased anxiety and altered yohimbine and NE levels in plasma, whereas plasma NE levels are positively related to increased brain arousal in these cases (129). Targeting corticotropin-releasing factor receptor type 1 (CRF-R_1_) was also an option for treating active IBS patients. The NE pathway of the locus ceruleus complex is changed in patients with IBS, and CRF-R_1_ may reduce the responsiveness to stress (130).

At the same time, tryptophan metabolism is also related to depression in patients with IBS (131). The immune-sensitive enzyme indoleamine 2,3-dioxygenase (IDO), which is responsible for the degradation of tryptophan was increased in IBS, however, the level of neuroprotective kynurenic acid (KynA) and the ratio of KynA/Kyn were reduced (132). Riluzole is a glutamate uptake activator that modifies visceral hypersensitivity in adult stressed animals, but it does not influence adolescent animals (133). The results from clinical and animal experiments suggest that N-methyl-D-aspartate (NMDA) receptors play an important role due to their blockade, which reduces the negative effects of stress and anxiety (134, 135). These findings suggested that regulation of the Kyn/tryptophan pathway may affect the receptors of NMDA in the CNS, might be involved in the progression of depression and may be a therapeutic target against the psychotic syndrome of the IBS patients (136, 137). Another study has also shown that microinjection of different doses of glutamate into the hypothalamic paraventricular nucleus (PVN) can not only reduce visceral sensitivity but also reduce the frequency of vagus nerve discharge (138). In addition, it has been reported that compared to the control sample, mGluR7 gene and protein expression levels in the colonic mucosa of rats with visceral hypersensitivity were upregulated. However, the administration of AMN082 (an mGluR7 agonist) can reduce visceral hypersensitivity, which indicates that targeting mGluR7 may be useful for relieving IBS (139).

## Conclusion and Future Perspective

Currently, the management of IBS has attracted attention due to the lack of effective medication and the difficulty of single-agent-based therapy to relieve symptoms. Intestinal microflora disorders are found in patients with different subtypes of IBS. At present, the gut microbiota is closely associated with IBS onset and symptoms in multiple aspects. Although probiotics have achieved preliminary efficacy in the treatment of IBS, the therapeutic mechanism remains unclear. IBS patients with neurotransmitter dysfunction have a series of symptoms, such as disturbance of the intestinal environment. Increasing evidence has proven that bidirectional communication exists between the gut microbiota and neurotransmitters. Neurotransmitters participate in blood flow, the absorption of nutrients, the gut microbiota, immunity, and intestinal movement to control and maintain the balance of the intestinal environment. Exploring its potential functions will help to understand the pathophysiology of IBS and find new targets for the treatment of IBS. However, the gastrointestinal tract is a complex system controlled by multiple regulators. The hormones produced by local mediators, the CNS, ENS, and other organs will affect the concentrations of neurotransmitters and their ultimate impact on intestinal physiology. Many studies have reported that 5-HT and GABA interact with the gut microbiota in many studies of functional gastrointestinal diseases, but relatively few studies have examined histamine and dopamine. This suggests that neurotransmitters need to be given more attention in future studies of the microbiota-gut-brain axis. Intestinal microbes can produce neurotransmitters and regulate them along the gut-brain axis. Therefore, more efforts are needed to reveal the mechanism of the microbiota-gut-brain axis in IBS. The impact of the microbiota composition on modulating neurotransmitter signals along the microbiota-gut-brain axis opens up an innovative and interesting approach. Animal studies that combine microbiota intervention with neurotransmitter receptor antagonists support this idea more strongly. However, most of the evidence was based on animal experiments, and thus there is an urgent need for a well-designed clinical study to verify this. This review provides a basis to continue the exploration of the complex interactions between neurotransmitters and their receptors, and the microbiota-gut-brain axis in the pathophysiology of IBS.

## Author Contributions

Conceptualization: YW and YC. Investigation: MC, GR, SY, LC, FX, ZX, GL, YT, LL, and YP. Writing—Original Draft Preparation: MC, GR, and LC. Writing—Review and Editing: YC and MC, and GR. Supervision: YW. All authors contributed to the article and approved the submitted version.

## Funding

This work was funded by grants from the Army Medical University Project (2017XYY06), Chongqing Science and Health Joint Project (2019ZDXM026), Army Medical Center Military Medical Frontier Innovation Capability Program (2019CXJSB008).

## Conflict of Interest

The authors declare that the research was conducted in the absence of any commercial or financial relationships that could be construed as a potential conflict of interest.

## Publisher’s Note

All claims expressed in this article are solely those of the authors and do not necessarily represent those of their affiliated organizations, or those of the publisher, the editors and the reviewers. Any product that may be evaluated in this article, or claim that may be made by its manufacturer, is not guaranteed or endorsed by the publisher.
